# Influence of Charged Self-Assembled Monolayers on Single Nanoparticle Collision

**DOI:** 10.1021/acs.analchem.2c04081

**Published:** 2023-01-26

**Authors:** Linoy Dery, Shahar Dery, Elad Gross, Daniel Mandler

**Affiliations:** † Institute of Chemistry, The Hebrew University, Jerusalem 91904, Israel; ‡ The Center for Nanoscience and Nanotechnology, The Hebrew University, Jerusalem 9190401, Israel

## Abstract

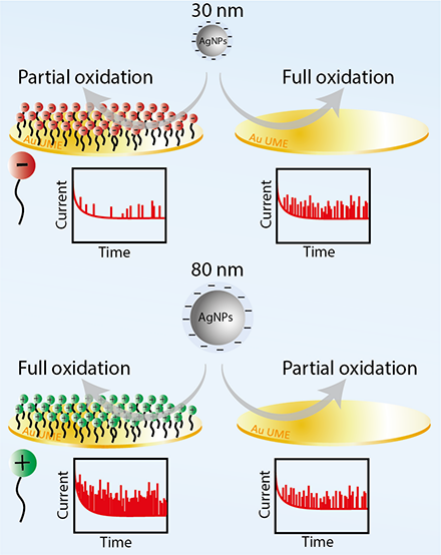

Studying nanoparticle (NP)–electrode interactions in single nanoparticle collision events is critical to understanding dynamic processes such as nanoparticle motion, adsorption, oxidation, and catalytic activity, which are abundant on electrode surfaces. Herein, NP–electrode electrostatic interactions are studied by tracking the oxidation of AgNPs at Au microelectrodes functionalized with charged self-assembled monolayers (SAMs). Tuning the charge of short alkanethiol-based monolayers and selecting AgNPs that can be partially or fully oxidized upon impact enabled probing the influence of attractive and repulsive NP–electrode electrostatic interactions on collision frequency, electron transfer, and nanoparticle sizing. We find that repulsive electrostatic interactions lead to a significant decrease in collision frequency and erroneous nanoparticle sizing. In stark difference, attractive electrostatic interactions dramatically increase the collision frequency and extend the sizing capability to larger nanoparticle sizes. Thus, these findings demonstrate how NP–monolayer interactions can be studied and manipulated by combining nanoimpact electrochemistry and functionalized SAMs.

## Introduction

Nanoimpact electrochemistry (NIE), also known as single particle collision, is a unique approach for studying nanomaterials at electrified surfaces.^1−6^ NIE allows breaking the pattern of the ensemble and gaining knowledge on the properties of single entities ranging from nanoparticles (NPs) to biological entities such as viruses, bacteria, and enzymes.^7−11^ The wealth of physical parameters that can be extracted from NIE includes, NP size distribution,^12^ porosity,^13^ concentration,^14^ particle agglomeration,^15^ and catalytic activity.^16,17^ In this respect, anodic particle coulometry (APC) first introduced by Compton et al. is the most widely used technique to acquire these analytical data on metallic NPs.^12^

In APC, stochastic collisions of single metallic NPs, typically Ag nanoparticles (AgNPs), result in their direct electrolysis due to a positive oxidative potential at the microelectrode surface.^12,14^ The oxidative electrolysis process is monitored by tracking single spikes in a chronoamperogram. Accurate counting and integration of the spikes afford a straightforward detection of NP size distribution and concentration.^12,14^ Over the last decade, considerable efforts have been devoted to studying the dynamic nature of stochastic impacts.^1^ For instance, it was demonstrated that a single collision of AgNPs does not necessarily lead to full electrolysis, or in other words, a large enough AgNP may experience incremental oxidation in a repeated multicollision process.^18−21^

These observations have set the stage for more recent works that have focused on how NP–electrode interactions can be controlled and manipulated.^3,22−24^ For example, Long’s group has explored the venue of dynamic NP–electrode interactions by tuning the adsorptive interactions on a Au ultramicroelectrode.^25^ In their report, AgO*_x_* NPs displayed diminished multipeak collisions and produced more uniform NP oxidation peaks in alkaline media. Consequently, high-resolution size distribution measurements of AgNP mixtures were successfully demonstrated.^25^ A similar concept was employed by Zhang et al. that used an ultrathin polysulfide adhesive layer and a thiosulfate electrolyte as a Lewis base to promote the oxidative dissolution of AgNPs.^26,27^ By enhancing the sticking probability of AgNPs to the microelectrode surface, their unique setup yielded an increase in collision frequency and accurate sizing measurements for 80 and 100 nm AgNPs.^27^ Nevertheless, these important reports used auxiliary processes rather than studying and improving the direct oxidation of Ag to its cationic form.

Inspired by these contributions, we aimed to elucidate the influence of the electrode’s surface charge on the NP–electrode electrostatic interactions. Negatively and positively charged as well as neutral functionalized short aliphatic thiols were used for the formation of self-assembled monolayers (SAMs) at the Au microelectrode surface, furnishing a defined surface charge at the interface. In this fashion, our approach circumvents the use of alkaline medium or a thiosulfate electrolyte and enables a facile probing of the NP–electrode electrostatic interactions. Notably, the use of charged SAMs for probing NP–electrode interactions has been introduced by Unwin^28^ and co-workers to investigate the interaction between citrate-capped AuNPs and an alkanethiol-modified Au electrode using scanning electrochemical cell microscopy. Moreover, Bard^29^ and Crooks^30^ used thiol-based SAMs to study electrocatalysis during single Pt NP collisions. However, these studies have not probed NP–electrode electrostatic interactions through the periscope of charged SAMs in APC studies. To the best of our knowledge, only a single report by Wolfrum et al. tried to address this challenge.^31^ Nevertheless, in that report, relatively long functionalized alkanethiols were used, and therefore, the influence of the hydrophobic chains was not decoupled from that of the charged functionalized groups.

In this work, APC studies were conducted using negatively charged citrate-capped AgNPs at the surface of a Au microelectrode functionalized with charged and short alkanethiol-based SAMs. These studies reveal that NP–electrode electrostatic interactions have a significant influence on both the collision frequency and sizing of AgNPs. By careful selection of 30, 55, and 80 nm AgNPs, an “oxidation spectrum” was formed ranging from complete to partial oxidation for the 30 and 80 nm AgNPs, respectively. We were able to meticulously study the influence of electrostatic interactions at each end of the spectrum. Repulsive NP–electrode electrostatic interactions resulted in a substantial decrease in collision frequency, whereas attractive electrostatic interactions increased them dramatically. The influence of NP–electrode electrostatic interactions on collision frequency was found to be size-independent between 30 and 80 nm NPs, and thus, in principle, the electrostatic attraction can be harnessed for APC studies in highly diluted solutions.

An additional remarkable observation was the effect of NP–electrode electrostatic interactions on electron transfer kinetics upon impact. Repulsive electrostatic interactions led to decreased electron transfer kinetics and erroneous sizing of AgNPs, regardless of their diameter. In contrast, attractive electrostatic interactions improved electron transfer kinetics at the interface and enabled the sizing of 80 nm AgNPs, which was previously hard to attain.

## Experimental Section

### Chronoamperometry Experiments (Nanoimpact)

Electrochemical dissolution was conducted in a solution containing varied AgNP concentrations, 10 mM KBr and 2 mM trisodium citrate as a stabilizer. A constant potential of +0.2 V was applied to the working electrode (100 μm diameter microelectrode), and Ag/AgCl and Pt wire were used as a QRE and counter electrode, respectively. All solutions were used for 15 min max to avoid aggregation.

### Electrode Functionalization

Au surfaces or microelectrodes were immersed in an ethanol solution containing 10 mM cysteamine, 3-mercaptopropionic acid, or 1-pentanethiol for 24 h. Then, the electrodes or surfaces were washed with ethanol and dried before use.

## Results and Discussion

Anodic stripping voltammetry was conducted to identify the onset oxidative potential for AgNP electrolysis.^12^ A 100 μL solution of 2 μg/mL 55 nm diameter AgNPs was drop-casted on the surface of a 3 mm diameter Au electrode. Next, linear sweep voltammetry (LSV) was performed in 0.1 M KBr solution. An oxidative striping is observed at −35 mV vs Ag/AgCl (Figure S1), and therefore, to ensure that the applied potential is not a limiting factor, an oxidative potential of 200 mV vs Ag/AgCl was used for the following APC experiments. The selection of the KBr electrolyte was carefully planned as Compton et al. demonstrated that AgNP impact frequency increases when potassium halide (Cl^–^, Br^–^, and I^–^) electrolytes are used as compared with a standard KNO_3_ solution.^31^ This increase was associated with the faster kinetics of the AgNP oxidation process in the presence of the halide anions that lead to a change in impact frequency. Additionally, AgNPs are only stable in a relatively narrow electrolyte concentration, which impedes our ability to test screening effects across a wide range of concentrations. In this manner, our experiments are not constrained by the kinetics of the oxidation process and can directly capture the effect of NP–electrode electrostatic interactions.

**Figure 1:**
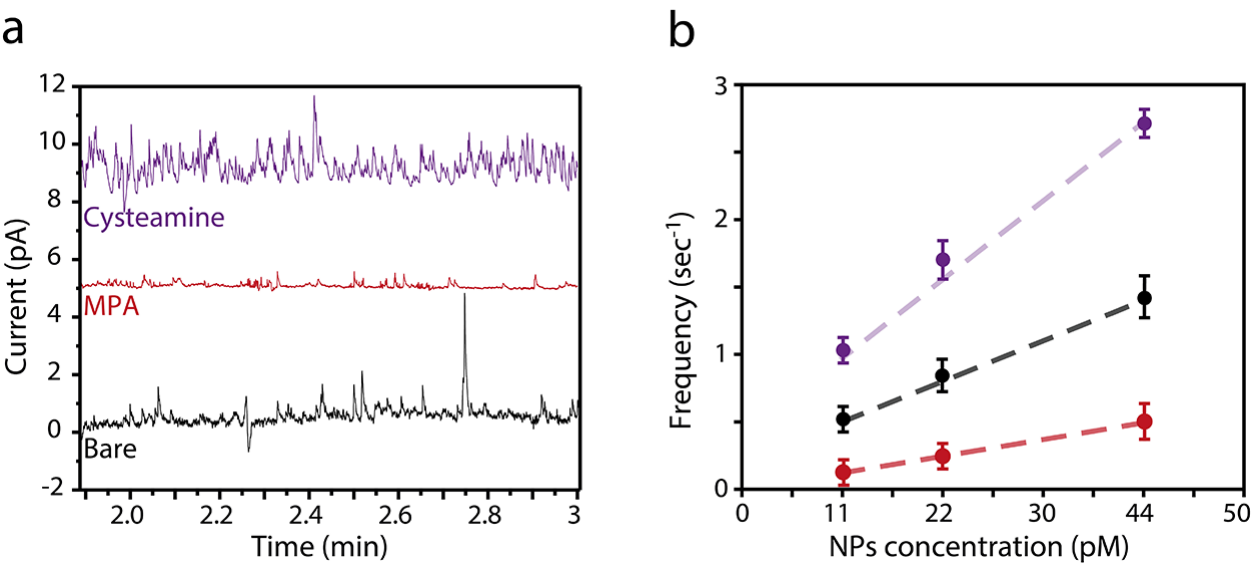
(a) Current–time traces for 30nm AgNP collisions at bare (black) and MPA (red)- and cysteamine (purple)-modified Au microelectrodes (in solution of 44 pM AgNPs). (b) Plot of the collision frequency at the different substrates vs AgNP concentration. All experiments were conducted at a sampling rate of 5 ms and an applied potential of 0.2 V vs Ag/AgCl quasi-reference electrode. Pt wire was used as the counter electrode. Each solution contained 10 mM KBr and 2 mM trisodium citrate.

Initial APC experiments were conducted in 11, 22, and 44 pM solutions of citrate-capped 29 ± 5 nm diameter AgNPs (calculated from transmission electron microscopy (Figure S2)) at an electropolished 100 μm diameter Au microelectrode (Figure 1a). NP concentrations are reported in pM (AgNPs per liter) using the mean average diameter of the NPs as obtained from the TEM images (see the Supporting Information). The purpose of this control experiment was to establish a basis collision frequency for the 29 ± 5 nm AgNPs. The black current–time trace in Figure 1a depicts such an experiment in which a solution of 44 pM AgNPs was used. Analysis of the spikes in the current–time trace displays a collision frequency of 1.3 s^–1^.

Next, the positively charged, cysteamine-functionalized Au microelectrode was studied under identical solution conditions. Analysis of its current–time trace reveals a 2-fold increase in the collision frequency to 2.7 s^–1^. The significant increase in the collision frequency suggests that the positively charged monolayer has a dominant influence on the electrode surface, irrespective of the positive potential that is applied throughout the measurement. This additional positive charge at the interface leads to a stronger attractive electrostatic interaction that favors the negatively charged citrate-capped AgNPs. In stark difference, the negatively charged 3-mercaptopropionic acid (MPA)-functionalized Au microelectrode yielded a collision frequency of 0.5 s^–1^, which is nearly a 3-fold decrease from that of the bare Au microelectrode. Further corroborating our hypothesis for the cysteamine SAM, the dramatic decrease in collision frequency for MPA demonstrates the effect of repulsive electrostatic interactions. These repulsive electrostatic interactions overcome the applied positive potential at the microelectrode surface and lead to an “electrostatic barrier” that hinders the motion of AgNPs toward it.^28,30^ It should be mentioned that, for the cysteamine-modified electrode at high AgNP concentrations, the impact frequency analysis is not trivial since some of the peaks are not fully separated. Thus, to correctly evaluate the impact frequency at these high concentrations, the full width at half-maximum (FWHM) that was measured for the oxidation peaks at low concentration was taken as the standard width.

APC studies with lower AgNP concentrations by 2- and 4-fold to 2 and 1 μg/mL, respectively, were conducted for the bare and MPA- and cysteamine-functionalized Au microelectrodes (Figure 1b). Markedly, the collision frequency for all of the studied concentrations displayed the following trend cysteamine > bare > MPA, which is in line with the expected electrostatic interaction trend moving from attraction to repulsion. Furthermore, a linear increase in collision frequency was observed for all of the studied surfaces along the concentration gradient; the linear trend is in accordance with previous results.^30,32^

The highest slope value was obtained for the cysteamine-functionalized Au microelectrode, an additional indication for the effectiveness of the attractive electrostatic interaction. Contrastingly, the lowest slope value, 5-fold lower than for cysteamine, was observed for the MPA-functionalized electrode. In essence, the higher collision frequencies obtained for the attractive NP–electrode electrostatic interactions can be exploited to increase the sensitivity of APC experiments to highly diluted solutions.

**Figure 2:**
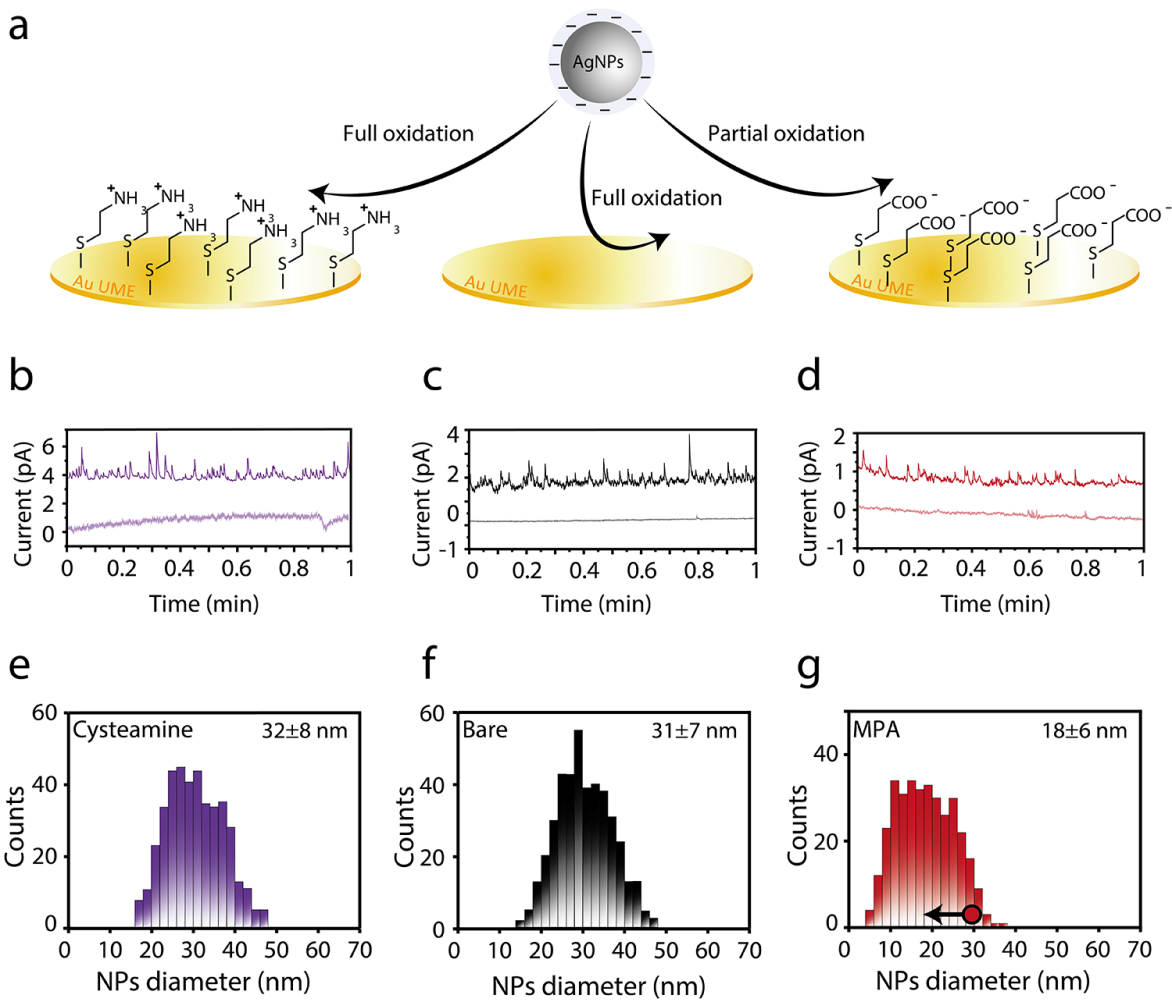
(a) Schematic illustration of the APC studies conducted on the functionalized Au microelectrodes. (b–d) Current–time traces of 30 nm AgNP collisions on cysteamine (22 pM)-functionalized, bare (22 pM), and MPA (44 pM)-functionalized Au microelectrodes, respectively. The lower curve represents the blank measurement without AgNPs. (e–g) Corresponding distribution of integrated current transients calculated from the current–time traces. All experiments were conducted at a sampling rate of 5 ms and an applied potential of 0.2 V vs Ag/AgCl quasi-reference electrode. Pt wire was used as the counter electrode. Each solution contained 10 mM KBr and 2 mM trisodium citrate.

A clear manifestation of the NP–electrode electrostatic interactions was demonstrated following the integration of the spikes for the different current–time traces (Figure 2e–g). The size distributions of 30 nm AgNPs were calculated by integrating each spike to afford the transferred charge. Next, the diameter of each NP associated with each spike was calculated using the typical equation assuming a spherical NP^12^ (see the Supporting Information (SI)). This analysis afforded a mean particle size of 31 ± 7 nm for the bare Au microelectrode and 32 ± 8 and 18 ± 6 nm for the cysteamine- and MPA-functionalized Au microelectrodes, respectively. The similar size distribution determined for the bare Au microelectrode supports the aforementioned results obtained from the TEM analysis. AgNPs (30 nm) are known to undergo full oxidation within a single collision,^18,19,27^ and thus, the accurate sizing for the bare Au microelectrode is in line with previous results.^25^ Similarly, the positively charged cysteamine-coated Au microelectrode displayed nearly identical size distribution indicating that the attractive interactions are less pronounced when complete oxidation is expected.

The erroneous sizing for MPA is a direct observation of the influence of electrostatic repulsion as it indicates that electron transfer inside the tunneling region is inefficient. Two different scenarios can explain the observed phenomenon:^25,27,33^ (1) the residence time of AgNPs within the tunneling region is too short or (2) the movement trajectory inside the tunneling region is limited, i.e., large distance from the electrode prevents efficient electron transfer. Both scenarios, which may occur simultaneously, will inevitably lead to incomplete oxidation of AgNPs at the Au microelectrode surface.

As an additional control, 1-pentanethiol, a neutral thiol spacer, was used as a representative for a hydrophobic thiol-based SAM bearing a neutral surface charge. APC experiments conducted with the 1-pentanethiol-coated Au microelectrode in a 44 pM solution of 30 nm AgNPs reveal a collision frequency of 0.8 s^–1^ and mean particle size of 30 ± 7 nm (Figure S3). The small decrease in collision frequency from the clean Au microelectrode is attributed to a densely packed monolayer (see below XPS) and to the fact that 1-pentanethiol has three additional methylene groups (∼1 Å per carbon atom) that act as a spacer impeding electron transfer.^29^ These results are in line with recent AgNP collision studies on SAM-modified Au microelectrodes.^22^ The comparable mean particle size obtained for 1-pentanethiol suggests that electron transfer upon impact is similar in its efficiency to that of a bare Au surface. These results further strengthen the notion that electrostatic repulsion invoked by the negatively charged SAMs has a dominant influence on electron transfer within the tunneling region.

To further support this claim, cyclic voltammetry with hexacyanoferrate(III) as a redox couple was performed with a clean Au electrode and following thiol adsorption (Figure S4). Cysteamine and 1-pentanethiol functionalizations show a negligible influence on the CV response as compared with a bare Au electrode indicating efficient electron transfer prior to and following thiol adsorption. However, for MPA, a clear decrease in the current is observed due to the repulsive interaction between the hexacyanoferrate(III) anion and the negatively charged MPA monolayer. This electrostatic trend is well established for small-molecule redox couples^28,30^ and clearly supports the electrostatic trend observed here for NPs.

X-ray photoelectron spectroscopy (XPS) measurements of the thiol-functionalized electrodes were conducted to provide quantitative data on the surface coverage of the formed SAMs. As expected, the XP spectra of S_2p_ confirmed the formation of thiol-based SAMs (Figure S5) and, in a similar manner, the C_1s_ and N_1s_ spectra identified the presence of the carboxylic and amine groups of MPA and cysteamine, respectively.^34^ Analysis of the atomic concentration of sulfur was conducted to elucidate the SAMs’ surface coverage as sulfur can solely originate from these organic ligands. The relatively similar surface coverage of cysteamine- and MPA-coated electrodes indicates that the electrostatic interactions studied in our experiments are not biased or largely affected by differences in surface coverage. It should be noted that the lower surface coverage calculated for 1-pentanethiol is most probably due to attenuation of the S_2p_ photoelectrons by its extra methylene groups.^35^

Further APC studies were performed for 55 nm AgNPs to evaluate the influence of NP–electrode electrostatic interactions on the sizing capabilities of the modified Au microelectrodes. Moreover, 55 nm AgNPs represent the limit of successful oxidation upon a single impact, and hence, it was hypothesized that it will be an interesting milestone to study.^20,21^ Collision experiments in a 1.8 pM solution of 55 nm AgNPs at a bare and cysteamine-functionalized Au microelectrode provided a mean particle size of 55 ± 15 and 55 ± 10 nm, respectively (Figure S6). These results are in excellent accord with the value of 54 ± 10 obtained by TEM analysis (Figure S2).

On the other hand, inaccurate sizing of 45 ± 9 nm was achieved for the MPA-coated microelectrode from the transients recorded in the 55 nm AgNP solution. This implies on a repulsive NP–electrode electrostatic interaction between the MPA SAM and the citrate-capped AgNPs. In this manner, the sizing results obtained for 55 nm AgNPs corroborate the results presented above (Figure 2) for 30 nm. Additionally, a higher collision frequency was recorded for the cysteamine-modified microelectrode as compared with bare or MPA-modified microelectrodes for the 55 nm AgNPs (Figure S7). This result indicates a similar influence of surface functionality on collision frequency. Taken together, these results demonstrate the dominance of the electrostatic repulsion at the regime where complete electrolysis of the Ag nanoparticle is expected.^12^

In 2017, the groups of Long,^20^ Zhang,^18^ and Unwin^21^ independently reported on a limitation of APC studies in AgNPs that are larger than ∼50 nm in diameter. These works pointed to the incomplete oxidation of large nanoparticles upon impact, and mechanistic investigations revealed multicollision patterns that were attributed to the bouncing of NPs following an initial impact. In light of these reports, 80 nm AgNPs were intentionally chosen as prime candidates for examining the influence of attractive NP–electrode electrostatic interactions on NP sizing. APC experiments performed on a bare and cysteamine-modified Au microelectrode in a 0.6 pM solution of 80 nm AgNPs unveiled that the attractive electrostatic interaction dominates the regime in which partial oxidation is predicted.

**Figure 3:**
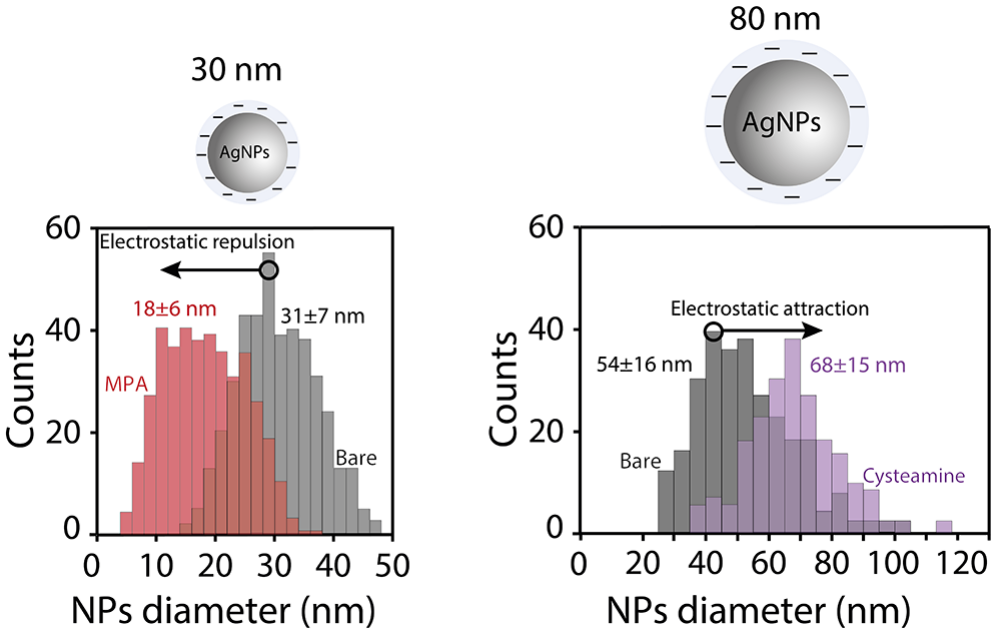
Corresponding distribution of integrated current transients calculated from the current–time traces of 30 and 80 nm AgNPs (left and right panels, respectively). The bare Au microelectrode is color-coded in gray, whereas MPA- and cysteamine-functionalized Au microelectrodes are color-coded in red and purple, respectively.

Integration of the spikes in the current–time trace in Figure S7 reveals a mean particle size of 54 ± 16 and 68 ± 15 nm for the bare and cysteamine-functionalized Au microelectrode, respectively (Figure 3, right panel). The former value implies incomplete oxidation of the AgNPs, which according to the TEM analysis displayed a mean particle size of 75 ± 10 nm (Figure S2). APC experiments on the MPA-modified Au microelectrode were not performed since even for the bare microelectrode, the 80 nm AgNPs did not fully oxidize. Importantly, this incomplete oxidation on a bare Au microelectrode is in agreement with previous reports.^25,27^

Collision frequency analysis of the 80 nm AgNPs was conducted at different AgNP concentrations for the bare and cysteamine-functionalized microelectrode (Figures S8 and S9) to further elucidate the influence of attractive electrostatic interactions. This analysis revealed a significantly higher collision frequency for the cysteamine-functionalized microelectrode as compared with the bare Au microelectrode. Essentially, the collision frequency for the cysteamine-modified electrode was overly high and therefore 5-fold lower AgNP concentrations were used. This analysis is in line with the results obtained for 30 nm AgNPs, indicating the robust influence of the cysteamine monolayer in shaping the attractive NP–electrode electrostatic interaction.

Moreover, the significant positive shift in mean particle size by 14 nm that is observed for the cysteamine-coated Au microelectrode constitutes a strong indication of the influence of attractive electrostatic interactions. In fact, the calculated sizes obtained by TEM analysis and APC experiments are well within the error margin of the two values. Several previous reports have discussed the inclination of the two-dimensional (2D) projection method to overestimate the volume of large NPs (>50 nm) in TEM.^27,36^ An overestimation of up to 18% was witnessed for these larger NPs due to heterogeneities in morphology, faceting, and defects.^36^ Therefore, we reason that our single collision studies do provide accurate sizing for larger NPs.

The observed shift in size distribution for the cysteamine SAMs is comparable with that of MPA (Figure 3, left panel), 11 and 13 nm, respectively. Two possible pathways are suggested as an explanation for the attractive NP–electrode electrostatic interactions: (1) electron transfer is more efficient upon a single impact and (2) the nanoparticle dwells longer within the tunneling region.

One way to discern between the two would be to perform high temporal resolution experiments similar to previous works.^12,18−21^ However, we were unable to achieve this temporal resolution since 100 μm gold electrodes were used in this study to obtain high surface density and close packing of the functionalized thiols. This was essential to minimize possible effects from uneven coating or defects at the interface between the electrode and its insulation sheet.

**Figure 4:**
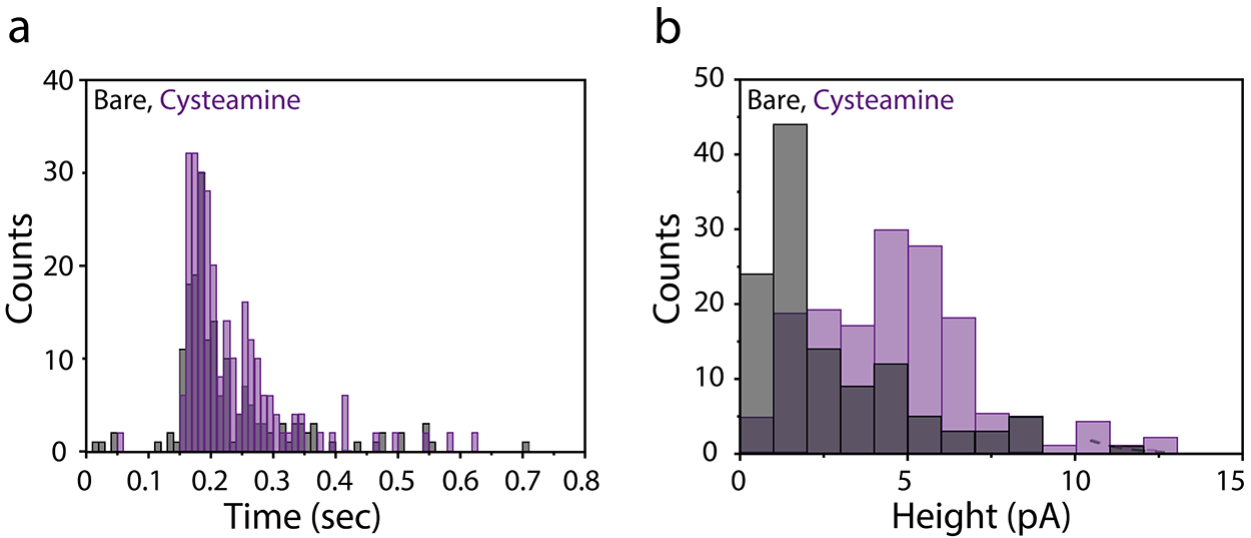
Histograms displaying the (a) full width at half-maximum and (b) height of the current transients obtained from the current–time traces of bare and cysteamine-functionalized Au microelectrodes in a solution of 80 nm AgNPs.

As an alternative way to try and discern between the abovementioned scenarios, two straightforward analyses are presented, full width at half-maximum (FWHM) (Figure 4a) and height of the peaks (Figure 4b) obtained from the current–time traces of the 80 nm AgNPs recorded with a bare and cysteamine-coated Au microelectrodes. Intriguingly, the FWHM histograms for both bare and cysteamine-functionalized Au microelectrodes fit nearly seamlessly, whilst the peak height shows a clear difference. Mean peak heights of 2.7 ± 2.3 and 4.7 ± 2.3 pA were obtained for the bare and cysteamine-coated Au microelectrodes, respectively. These results signify that the residence time inside the tunneling region is comparable, whereas the initial oxidation burst, as indicated by the peak height, is higher for the attractive electrostatic interaction invoked by cysteamine. Therefore, we deduce that the higher mean peak height for the latter is mainly associated with improved electron transfer kinetics upon an initial impact. Since electron transfer is highly dependent on the NP–electrode distance, it is hypothesized that the citrate-capped AgNPs reach closer to the cysteamine-modified microelectrode surface as compared with that of bare Au.

## Conclusions

In conclusion, this study has demonstrated the influence of charged SAMs on single collision experiments of citrate-capped AgNPs. The use of short functionalized thiols decoupled the effect of the long alkyl chains and, as a result, facilitated the probing of NP–electrode electrostatic interactions in a single particle collision. The negatively charged MPA-functionalized Au microelectrode showed decreased collision frequency and inaccurate sizing of AgNPs. In a stark difference, the positively charged cysteamine-functionalized Au microelectrode exhibited higher collision frequency and accurate sizing, expanding the sizing capability to 80 nm AgNPs. This expansion eludes the direct modification of AgNPs, electrolytes, or the pH of the solution. In essence, these results express the importance of NP–electrode electrostatic interactions and demonstrate that a modification by charged SAMs is sufficient to manipulate the electrostatic interactions at the microelectrode surface. For the attractive NP–electrode interactions, such manipulation can be harnessed to study single NP collisions in highly diluted solutions or to ensure the accurate sizing of larger NPs. It should be emphasized that these electrostatic interactions strongly depend on additional parameters, such as the electrolyte concentration. Yet, the limited stability of the AgNPs through a wide range of the electrolyte concentration impedes our ability to properly examine this effect.

The simplicity of the approach suggests that it may be extended to additional compositions of NPs or electrode surfaces and our laboratory is currently pursuing this direction. It is envisioned that this study will open the way for additional inquiries into electrostatic interactions of charged SAMs with other single entities such as bacteria, viruses, and proteins that have rich surface chemistry.

## Supporting Information Available

Materials, instrumentation, additional experimental details of chronoamperometry experiments (nanoimpact), measurement description, electrode functionalization, and NP sizing by nanoimpact; linear stripping voltammetry (LSV) of drop-cast 55 nm AgNPs (Figure S1); TEM images of 30, 55, and 80 nm AgNPs (Figure S2); current–time traces of 30 nm AgNP collisions on the 1-pentanethiol-modified Au microelectrode (Figure S3); CV of 10 mM potassium hexacyanoferrate(III) on bare and functionalized Au electrodes (Figure S4); Au 4f, C_1s_, S_2p_, and N_1s_ XPS of the thiol-functionalized Au electrodes (Figure S5); size distribution taken from the current–time traces and collision frequency at a bare or functionalized Au microelectrode for 55 nm AgNPs (Figures S6 and S7); representative current–time traces of 80 nm AgNP collisions and collision frequency at at a bare or functionalized Au microelectrode for 80 nm AgNPs (Figures S8 and S9) (PDF)
