# Supplementary material for: Influence of Charged Self-Assembled Monolayers on Single Nanoparticle Collision
Source: Anal Chem. 2023 Jan 26;95(5):2789–95. doi: 10.1021/acs.analchem.2c04081 (PMC9909668; doi:10.1021/acs.analchem.2c04081)
Supplement: Supplementary file 1 — ac2c04081_si_001.pdf [file ac2c04081_si_001.pdf]

# Supplementary information

# The Influence of Charged Self-Assembled Monolayers on Single Nanoparticle Collision

Linoy Dery[1,2], Shahar Dery[1,2], Elad Gross[1,2], Daniel Mandler[1,2]*

1. Institute of Chemistry, The Hebrew University, Jerusalem 9190401, Israel
2. The Center for Nanoscience and Nanotechnology, The Hebrew University, Jerusalem 9190401, Israel

**Corresponding author**

* Daniel Mandler: daniel.mandler@mail.huji.ac.il

**Table of content:**

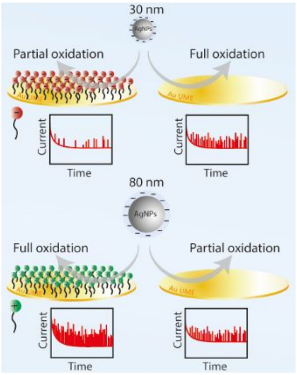


Self-assembled monolayers affect the sizing and electrode collision frequency of metallic nanoparticles measured by nano-impact electrochemistry

**Experimental section**

## Materials

Silver NPs (30, 50, and 80 nm) were purchased from nanoComposix, Inc. (San Diego, CA, USA. Potassium bromide, potassium chloride, and trisodium citrate dihydrate were purchased from Merck. Cysteamine, 3-mercaptopropionic acid. and 1-pentanethiol were obtained from Sigma-Aldrich. Potassium hexacyanoferrate(III) was purchased from Sigma. Ethanol (reagent grade) was purchased from J. T. Baker. Ultrapure deionized water (EasyPure UV, Barnstead) was used for all aqueous solutions.

## Instruments

**Electrochemical Measurements of single particle collision** were carried out using a homemade potentiostat that was fixed inside a Faraday cage to minimize the noise. This pA~nA current potentiostat (with high-speed data acquisition card extension) was used to study single entity electrochemistry in previous publications [Ionosomes: observation of ionic bilayer water clusters].

**X-ray photoelectron spectroscopy (XPS)** measurements were performed using Kratos AXIS Supra spectrometer (Kratos Analytical Ltd., Manchester, U.K.) with Al Kα monochromatic X-ray source (1486.6 eV). The XPS spectra were acquired with a takeoff angle of 90° (normal to analyzer); the vacuum condition in the chamber was $2 \times 10^{-9}$ Torr. High-resolution XPS spectra were acquired with a pass energy of 20 eV and a step size of 0.1 eV. The binding energies were calibrated according to the Au4$f_{7/2}$ XPS peak position (B.E. = 84.0 eV). Data were collected and analyzed by using ESCApe processing program (Kratos Analytical Ltd.) and Casa XPS (Casa Software Ltd.).

**Transmission electron microscopy (TEM)** images were taken in an aberration-corrected FEI Themis G3 instrument at 300 keV.

**Linear sweep voltammetry (LSV) and Cyclic Voltammetry (CV)** were conducted with a potentiostat (CHI-630, CH Instruments Inc.)

## Methods

All electrochemical measurements were performed in a Faraday cage at a temperature of 298 °K. The working electrodes were polished before using alumina powder 0.1 and 0.05 microns on a polishing pad. Then the electrodes were cleaned in water and sonication for 10 s. finally, electrochemical polishing was performed in 0.5 M $H_2SO_4$ solution from 0.2 to 1.6 V (vs. Ag/AgCl) until a repetitive CV was observed.

**NPs sizing by nano-impact** the charge of each spike was calculated by integration of the spike by Origin. Then, the diameter of each NP associated with each spike was calculated using the equation[1,2]:

$$D_{NP} = 2\sqrt[3]{\frac{3M_wQ}{4nF\pi\rho}}$$

Where $M_w$ is the molecular weight of silver (107.8 g/mol), Q is the charge, n is the number of electrons involved in the oxidation of one metal atom, F is the Faraday constant, and $\rho$ is the NPs density (10.49 g/cm$^3$ for Silver).

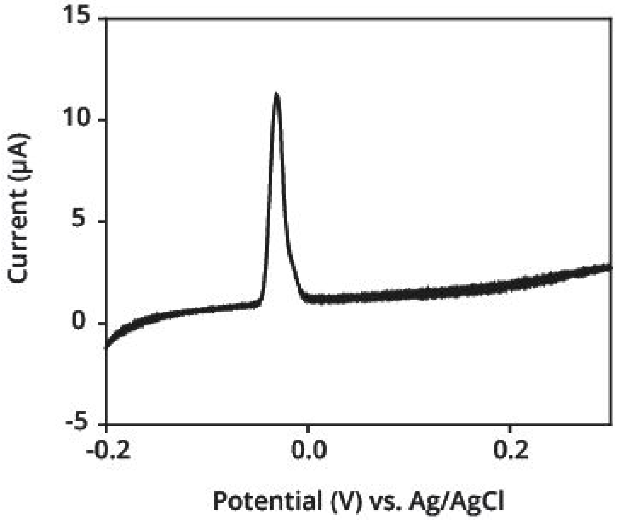


**Figure S1.** Linear stripping voltammetry (LSV) of drop-casted 55 nm AgNPs on Au electrode (3 mm diameter) in 0.1 M KBr solution at a scan rate of 0.1 V/sec. RE: Ag/AgCl and CE: Pt rod.

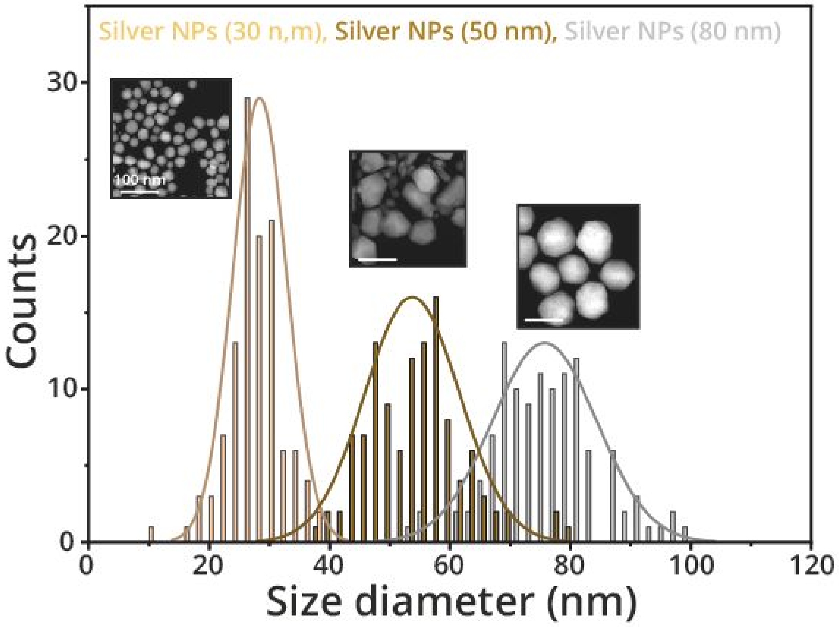


**Figure S2.** Transmission electron microscopy (TEM) images of 30, 55, and 80 nm AgNPs. The scale bar is 100 nm. Each size distribution was calculated from at least 200 NPs.

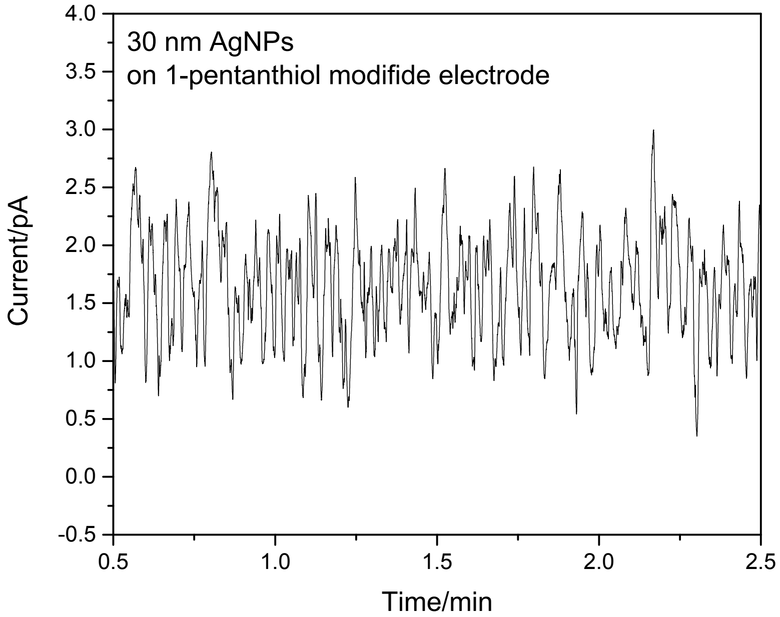


**Figure S3.** Representative current-time traces of 30 nm AgNPs collisions on 1-pentanethiol modified Au microelectrode. Potential of the microelectrode: +0.2 V. RE: Ag rod and CE: Pt rod.

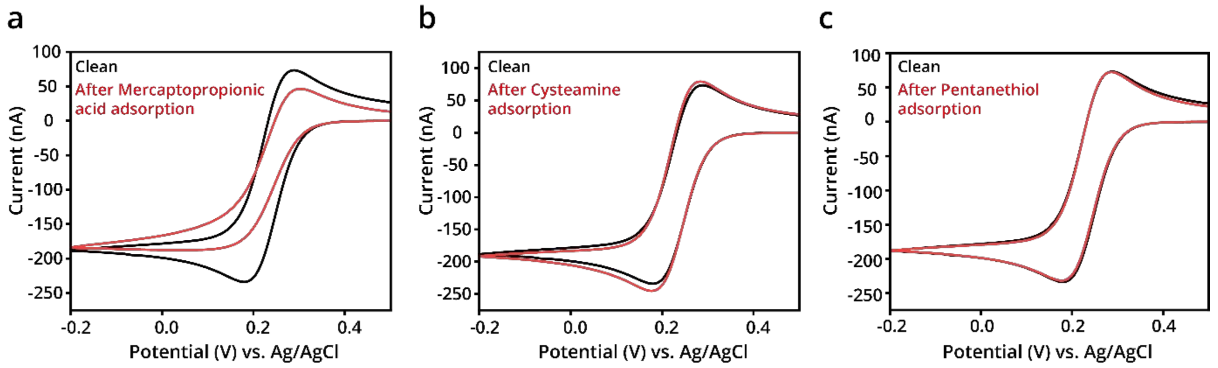


**Figure S4.** CV of 10 mM potassium hexacyanoferrate(III) on bare and functionalized Au electrodes: a. bare, b. cysteamine and c. MPA. All CVs were performed in 0.1 M KCl at a scan rate of 0.05 V/sec. RE: Ag/AgCl, CE: Pt rod.

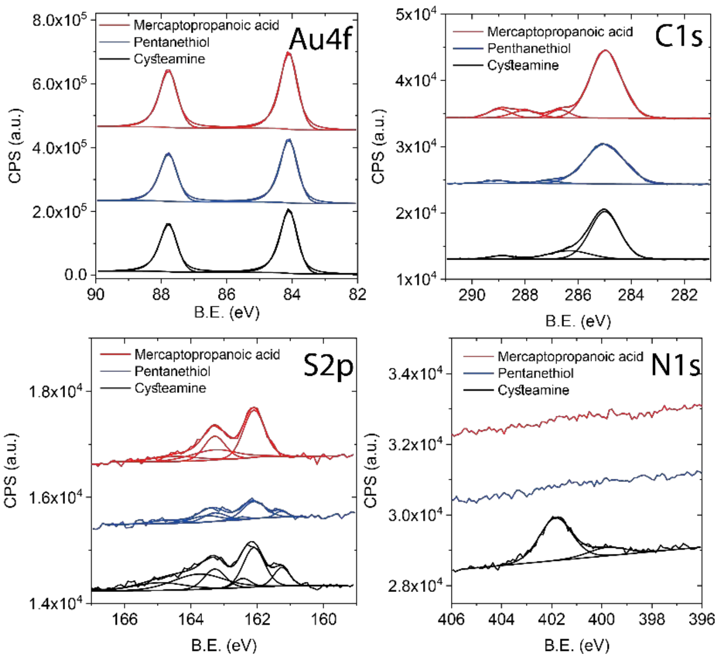


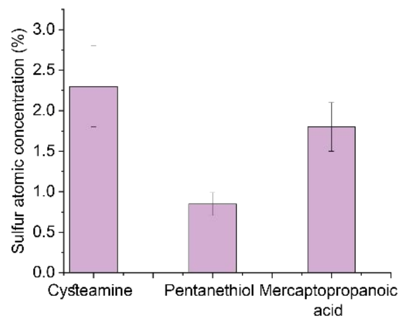


**Figure S5.** Au 4f, C 1s, S 2p, and N 1s XPS of the thiol-functionalized Au electrodes. Sulfur atomic concentration was analyzed based on the XPS data and normalized to the ionization cross-section of sulfur.

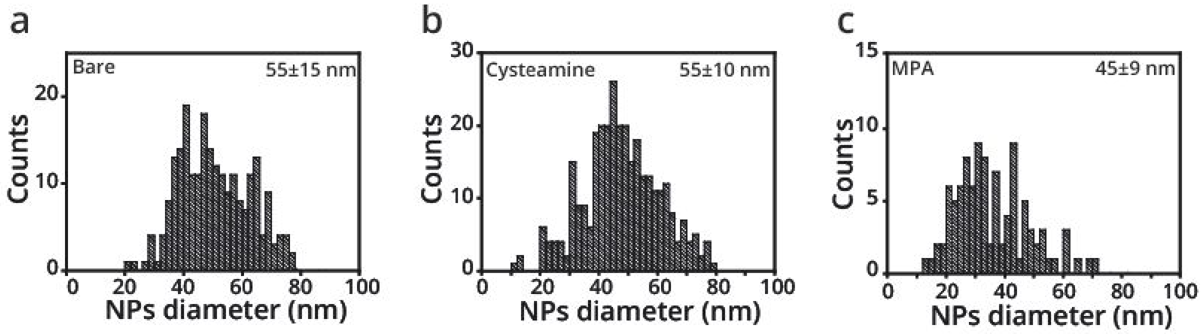


**Figure S6.** The corresponding size distribution of integrated current transients taken from the current-time traces of 55 nm AgNPs collisions at Au electrodes: a. bare b. cysteamine and c. MPA SAMs.

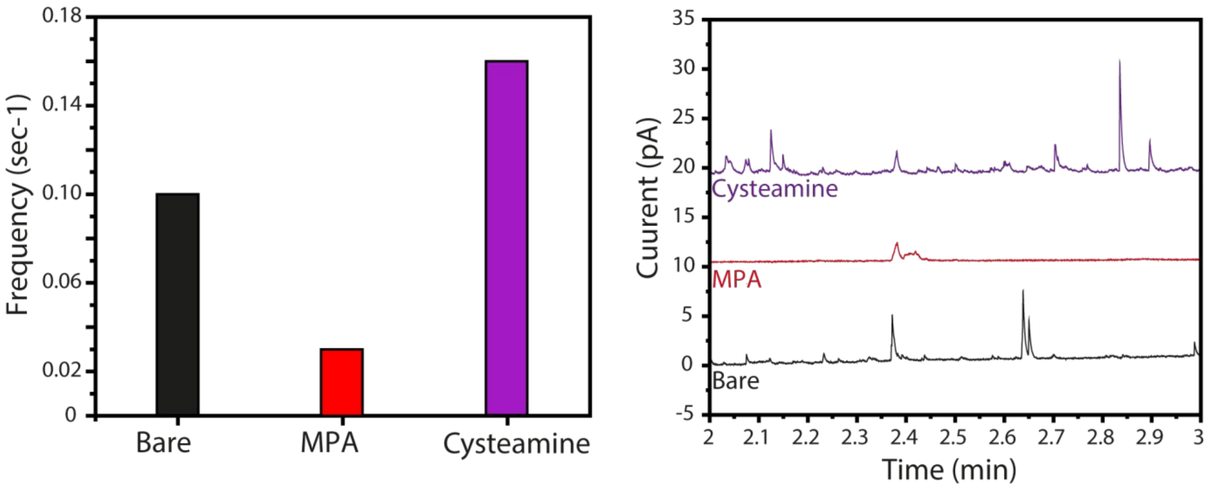


**Figure S7. Left:** Collision frequency of 55 nm AgNPs (1.8 pM) at bare or functionalized Au microelectrode. **Right:** Representative current-time traces of 55 nm AgNPs collisions on cysteamine (purple), bare (black), and MPA (red) functionalized Au microelectrodes, respectively. Potential of the microelectrode: +0.2 V. RE: Ag rod and CE: Pt rod.

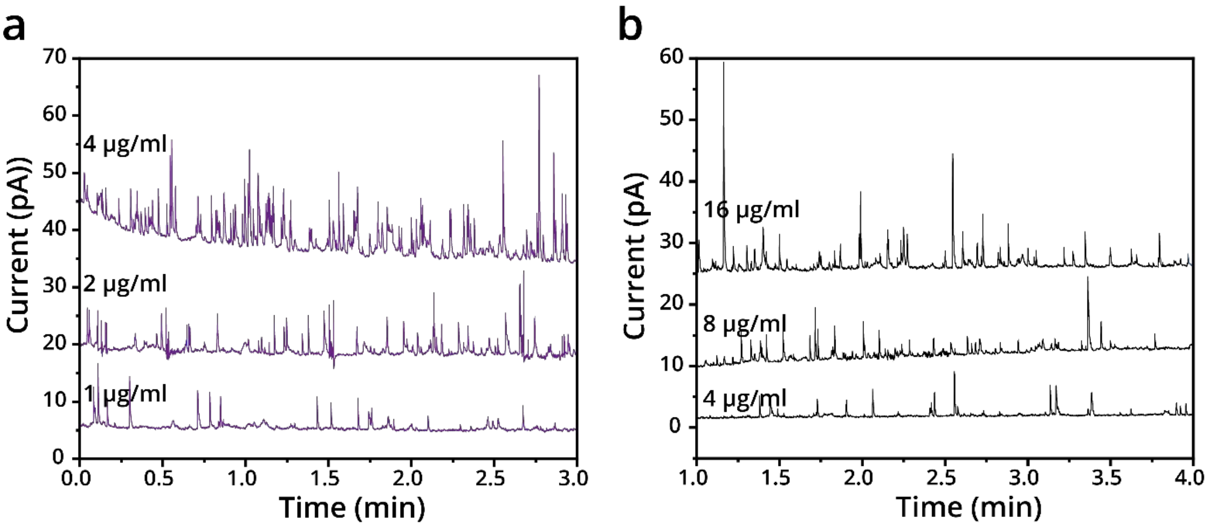


**Figure S8.** Representative current-time traces of 80 nm AgNPs collisions on cysteamine functionalized (purple) and bare (black) Au microelectrodes, respectively. Potential: +0.2 V. RE: Ag rod and CE: Pt rod.

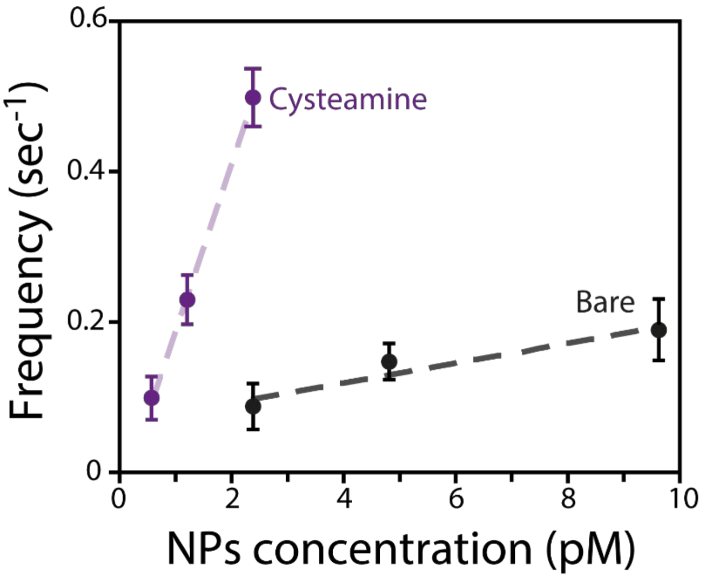


**Figure S9.** The collision frequency at the different substrates *vs.* AgNPs concentration for 80 nm Ag NPs. The collision frequency was calculated from the data presented in figure S7.

**References**

(1) Zhou, Y.-G.; Rees, N. V.; Compton, R. G. *Angewandte Chemie International Edition* 2011, *50*, 4219.

(2) Ellison, J.; Tschulik, K.; Stuart, E. J. E.; Jurkschat, K.; Omanović, D.; Uhlemann, M.; Crossley, A.; Compton, R. G. *ChemistryOpen* **2013**, *2*, 69.
